# Primary CD33-targeting CAR-NK cells for the treatment of acute myeloid leukemia

**DOI:** 10.1038/s41408-022-00660-2

**Published:** 2022-04-13

**Authors:** Nawid Albinger, Rita Pfeifer, Marcus Nitsche, Sarah Mertlitz, Julia Campe, Katja Stein, Hermann Kreyenberg, Ralf Schubert, Melissa Quadflieg, Dina Schneider, Michael W. M. Kühn, Olaf Penack, Congcong Zhang, Nina Möker, Evelyn Ullrich

**Affiliations:** 1 Childrens Hospital, Experimental Immunology, Johann Wolfgang Goethe University, Frankfurt am Main, Germany; 2 Frankfurt Cancer Institute, Johann Wolfgang Goethe University, Frankfurt am Main, Germany; 3 University Cancer Center (UCT) Frankfurt, Frankfurt am Main, Germany; 4 Miltenyi Biotec B.V. & Co. KG, Bergisch Gladbach, Germany; 5 Charité, Universitätsmedizin Berlin, corporate member of Freie Universität Berlin and Humboldt-Universität zu Berlin, Department of Department of Hematology, Oncology and Tumorimmunology, Berlin, Germany; 6 Division for Stem Cell Transplantation, Immunology, and Intensive Care Medicine, Department for Children and Adolescents, University Hospital, Johann Wolfgang Goethe University, Frankfurt am Main, Germany; 7 Childrens Hospital, Pulmology and Allergology, Johann Wolfgang Goethe University, Frankfurt am Main, Germany; 8 Lentigen Technology, Inc., a Miltenyi Biotec Company, Gaithersburg, MD USA; 9 Department of Hematology, Medical Oncology, and Pulmonary Medicine, University Medical Center, Johannes Gutenberg-University, Mainz, Germany; 10 German Cancer Consortium (DKTK), partner site Berlin and German Cancer Research Center (DKFZ), Heidelberg, Germany; 11 German Cancer Consortium (DKTK) partner site Frankfurt/Mainz and German Cancer Research Center (DKFZ), Heidelberg, Germany

**Keywords:** Translational research, Preclinical research

## Abstract

Acute myeloid leukemia (AML) is a malignant disorder derived from neoplastic myeloid progenitor cells characterized by abnormal proliferation and differentiation. Although novel therapeutics have recently been introduced, AML remains a therapeutic challenge with insufficient cure rates. In the last years, immune-directed therapies such as chimeric antigen receptor (CAR)-T cells were introduced, which showed outstanding clinical activity against B-cell malignancies including acute lymphoblastic leukemia (ALL). However, the application of CAR-T cells appears to be challenging due to the enormous molecular heterogeneity of the disease and potential long-term suppression of hematopoiesis. Here we report on the generation of CD33-targeted CAR-modified natural killer (NK) cells by transduction of blood-derived primary NK cells using baboon envelope pseudotyped lentiviral vectors (BaEV-LVs). Transduced cells displayed stable CAR-expression, unimpeded proliferation, and increased cytotoxic activity against CD33-positive OCI-AML2 and primary AML cells in vitro. Furthermore, CD33-CAR-NK cells strongly reduced leukemic burden and prevented bone marrow engraftment of leukemic cells in OCI-AML2 xenograft mouse models without observable side effects.

## Introduction

Acute myeloid leukemia (AML) represents a devastating disease for which only limited therapeutic progress has been made in the last decades. Besides high-dose chemotherapy, allogeneic bone marrow transplantation currently constitutes the most effective therapy, which is not applicable for all patients, often not fully eliminates tumor cells, and harbors the significant risk of inducing a graft-versus-host disease. Overall, this results in poor prognosis especially for elderly patients [1–4]. In other hematological malignancies such as B-cell acute lymphoblastic leukemia (B-ALL) novel cellular therapies with chimeric antigen receptor (CAR)-modified T cells lead to striking results which culminated in the approval of multiple drugs by the U.S. Food & Drug Administration (FDA) and the European Medicines Agency (EMA) [5–9]. Nevertheless, treatment of AML with CAR-T cells appears to be complicated due to the lack of AML-specific antigens and the tremendous molecular heterogeneity of the disease [1, 10]. While for B-ALL CD19 is well known as a highly specific antigen for leukemic blasts [11, 12], in AML such an exclusive antigen still needs to be identified. As a promising target CD33 was shown to be expressed on leukemic blasts and leukemia-inducing cells in the majority (88%) of AML patients, but also on normal hematopoietic stem cells [10, 13]. Furthermore, preclinical results demonstrated increased anti-AML activity of CD33-specific CAR-T cells [14, 15]. However, clinical application of long-persistent CAR-T cells appears to be challenging due to potential long-term suppression of hematopoiesis. Furthermore, CAR-T cells need to be generated from autologous sources, which is cost and time intensive and often not possible from heavily pre-treated patients. As adoptively transferred natural killer (NK) cells possess a shorter lifetime, are associated with fewer side effects, and hold an intrinsic CAR-independent killing capacity against AML, CD33-CAR-NK cells constitute a promising alternative [16–19]. The use of peripheral-blood-derived NK cells (PB-NK) also appears advantageous, since NK cell lines such as NK-92 need to be irradiated prior to infusion, which can limit the proliferation and killing capacity of applied cells [17]. In addition, NK cells do not induce graft-versus-host diseases. As a perspective, the allogeneic transfer of CAR-NK cells in combination with freezing of cells harbors the potential for a true “off-the-shelf” product, which could dramatically improve the availability and reduce the costs of such an immune-cell based therapy.

Here, we report on the generation of CD33-specific CAR-NK cells from peripheral blood using baboon envelope pseudotyped lentiviral vectors (BaEV-LV). CD33-CAR-NK cells showed stable CAR-expression, functional proliferation, and significantly increased killing capacity against CD33-positive AML cell lines as well as primary AML cells in vitro. Furthermore, CD33-CAR-NK cells effectively eliminated all bone marrow- and spleen-engrafted, as well as the majority of peripheral AML cells in an OCI-AML2 xenograft mouse model without detectable side effects.

## Materials and methods

### Primary AML cells

Frozen primary AML cells were thawed and cultivated in DMEM medium supplemented with 15% FBS, 50 µM ß-Mercaptoethanol, 1% Pen/Strep, 100 ng/mL stem cell factor (SCF), 10 ng/mL IL-3, 20 ng/mL IL-6, 10 ng/ml thrombopoietin (TPO) and 10 ng/ml FMS-like tyrosine kinase 3 ligand (FLT3L)). Cells were seeded with 700,000 cells in 3 ml per well of a six-well plate and 1 µg/ml f.c. DNAse was added. The usage of primary patient material was approved by the Ethics Commission of the University Hospital Frankfurt, Germany (approval no. 274/18). All participants gave written informed consent in accordance with the Declaration of Helsinki.

### Isolation of NK cells

This project was approved by the Ethics Committee of the Goethe University Frankfurt, Germany (approval no. 329/10 and 274/18). All participants gave written informed consent in accordance with the Declaration of Helsinki. NK cells were isolated from buffy coats of fresh blood from healthy, anonymous donors provided by the German Red Cross Blood Donation (DRK-Blutspendedienst Baden-Württemberg-Hessen, Frankfurt am Main, Germany) or from whole peripheral blood, acquired from healthy volunteer donors at Miltenyi Biotec (Bergisch-Gladbach, Germany). Peripheral blood mononuclear cells (PBMCs) were isolated by Ficoll density gradient centrifugation (Biocoll, Biochrom, Berlin, Germany). Subsequently, NK cell enrichment was performed by immunomagnetic selection of either CD3 depletion followed by an CD56 enrichment using MicroBeads (Miltenyi Biotec) or utilizing the EasySep™ Human NK Cell Enrichment Kit (StemCell, Vancouver, Canada) according to the manufacturer’s instructions. NK cell purity was determined by flow cytometry using fluorochrome-conjugated antibodies anti-CD56-APC (clone REA196, Miltenyi Biotec) or anti-CD56-BV421 (clone NCAM16.2, BD Biosciences, Franklin Lakes, New Jersey, USA), anti-CD3-BUV395 (clone SK7, BD Biosciences), anti-CD19-APC-Vio770 (clone REA675, Miltenyi Biotec) or anti-CD19-BB515 (clone HIB19, BD Biosciences), anti-CD20-BUV737 (clone 2H7, BD Biosciences), anti-CD45-VioBlue (clone REA747, Miltenyi Biotec) or anti-CD45-BV510 (clone HI30, BD Biosciences), anti-CD14-VioGreen (clone REA599, Miltenyi Biotec) or anti-CD14-BV711 (clone M5E2), as well as anti-CD16-PE (clone 3G8) (both Biolegend, San Diego, California, USA). Freshly isolated NK cells were cultured in NK MACS^®^ Medium (Miltenyi Biotec) supplemented with 5% heat-inactivated (h.i.) human plasma (DRK-Blutspendedienst), 1% NK-MACS^®^ Supplements (Miltenyi Biotec) and 1% Pen/Strep at concentrations of 1 × 10^6^ cells/ml and cells split every 3–4 days. Depending on the experiment additionally, 80 ng/ml IL-1β (Miltenyi Biotec), 500 IU/ml IL-2 (Novartis, Basel, Switzerland or Miltenyi Biotec), 10 ng/ml IL-15 (Peprotech, Cranbury Township, New Jersey, USA or Miltenyi Biotec) or 50 ng/ml IL-15 (CellGenix, Freiburg im Breisgau, Germany) was added [20, 21].

### CAR construction and lentiviral vector production

Second-generation CD33-CAR incorporating the My96 scFv sequence was constructed as described earlier [22]. Briefly, the My96 scFv was combined in frame with CD8 hinge and transmembrane domain, 4-1BB/CD137 co-stimulatory domain, and CD3ζ activation domain. A leader peptide derived from GM-CSFRα was included to facilitate CAR cell surface expression. Third-generation self-inactivating baboon envelope-pseudotyped lentiviral vectors (BaEV-LVs) were produced by transient transfection into HEK293T cells using MACSfectin (Miltenyi Biotec) or polyethylenimine (PEI) [23].

### Transduction of NK cells

On day two post NK cell isolation NK cells were transduced with lentiviral particles.

Therefore, 0.5 × 10^6^ cells per well were seeded in a flat bottomed 48-well plate. Subsequently, lentiviral particles and Vectofusin-1 (Miltenyi Biotec; 2.5 µg/ml final concentration per well) were mixed in identical volumes, incubated at room temperature for 7 min, and added to the cells, to reach a final cell concentration of 1 × 10^6^ cells/ml. All previous steps were performed in serum-free NK-MACS^®^ medium supplemented with 1% NK-MACS^®^ Supplements, with or without 1% Pen/Strep and 80 ng/ml IL-1β (Miltenyi Biotec), 500 IU/ml IL-2 (Miltenyi Biotec or Novartis). Finally, 10 ng/ml IL-15 (Miltenyi Biotec or Peprotech) or 50 ng/ml IL-15 (CellGenix) was added. Subsequently, the plate was centrifuged at 400 × *g* for 2 h at 32 °C. Twenty-four hours post-transduction half of the medium was replaced by fresh medium containing 5% human plasma and a combination of IL-2 and IL-15 [24].

### Flow cytometry analysis of transduced NK cells

CAR and CD16 expression on gene-modified NK cells as well as the possible contamination with CD3-positive cells were analyzed every 3–7 days post transduction using flow cytometry. For NK and CAR-NK cell phenotyping, fluorochrome-conjugated antibodies anti-KIR2D-VioBlue (clone REA1042), anti-CD16-VioGreen (clone REA423), anti-NKG2C-PE (clone REA205), anti-NKG2A-PE-Vio770 (clone REA110), anti-CD57-APC-Vio770 (clone REA769), anti-CD56-APC (clone REA 196), anti-NKp44-PE (clone REA1163), anti-NKp30-PE-Vio770 (clone REA823), anti-CD33-VioBright515 (clone REA775) (all Miltenyi Biotec), anti-DNAM-1-BV421 (clone 11A8), anti-NKG2D-BV510 (clone 1D11), anti-CD56-BV786 (clone NCAM16.2), anti-CD16-PE-CF594 (clone 3G8), anti-CD3-BUV395 (clone Sk7) (all BD Biosciences) were used. CD33-CAR expression was analyzed with a CD33-CAR Detection Reagent, containing a recombinantly expressed fusion protein consisting of the human CD33 extracellular domains and a specifically mutated human IgG1 Fc region (Miltenyi Biotec) and secondary addition of anti-biotin-PE antibody (clone REA746) (Miltenyi Biotec).

### CD33-expression analysis

CD33 expression on AML cell lines as well as primary AML and NK cells were analyzed by flow cytometry using anti-CD33-PE (clone REA775, Miltenyi Biotec) or -BV421 (clone WM53, BD Bioscience).

### CAR-NK cell functional assay

Four hours or 24 h endpoint cytotoxicity of CAR- and UTD-NK cells was analyzed by flow cytometry. Target cells either expressing GFP or stained with Cell Trace CFSE proliferation kit (Invitrogen) were co-cultured with effector cells at various effector-target (E:T)-ratios for the indicated time period at 37 °C and 5% CO_2_. Afterwards, cells were stained with 4′,6-diamidino-2-phenylindole (DAPI) (AppliChem), or propidium iodide (PI) (Miltenyi Biotec), and viability of target cells was analyzed using a BD FACSCelesta (BD Biosciences) or MACSQuant Analyzer 10 (Miltenyi Biotec).

To analyze cytotoxicity over time, real-time monitoring using the IncuCyte S3 system (Sartorius, Goettingen, Germany) was performed. Assays were set up by co-culturing GFP-transgenic target cells with CAR-NK cells at an E:T-ratio of 1:1 or 1:5. Phase contrast and green fluorescence images were captured with 10× magnification every two hours for 4 days. Analysis of images was performed using the following settings for GFP: Top-hat (110 μm) and Threshold (0.5 GCU).

Cytotoxicity upon repetitive tumor challenge was analyzed by co-incubating effector cells with GFP-expressing target cells at an E:T-ratio of 1:1 and rechallenging every 2 days with fresh target cells and 50% of new culture medium. Viable cell count was quantified by flow cytometry 24 h after each target cell addition. Cytokine secretion assays were conducted by co-culturing UTD- and CAR-NK cells with target cells at a ratio of 1:1 for 24 h. Harvested supernatants were then analyzed using the MACSPlex Cytokine 12 Kit for human analytes (Miltenyi Biotec) following manufacturer’s protocol.

### In vivo functional studies of CD33-CAR-NK cells in xenografted mice

NOD.Cg-*Prkdc*^*scid*^ *Il2rg*^*tm1Wjl*^ Tg(CMV-IL3,CSF2,KITLG)1Eav/MloySzJ (NSG-SGM3) mice were obtained from *The Jackson Laboratory*, Bar Harbor, ME, USA *(JAX stock No.: #013062 (NSG-SGM3))* [25]. Mice were held under standardized pathogen free conditions with adequate access to food and water. Experiments were approved by the Regierungspräsidium Darmstadt, Germany. To engraft tumor cells, 0.5 × 10^6^ OCI-AML2 (GFP^+^, Luc^+^) cells were injected via the tail vein into NSG-SGM3 mice (≤ 15 weeks old, male or female depending on the experiment). At day 2 post tumor cell application, mice were injected with luciferin subcutaneously and the bioluminescence signal was analyzed using an IVIS^®^ Lumina II Multispectral Imaging System (PerkinElmer, Waltham, MA, USA) to assess the tumor cell engraftment. On the following day, 1 × 10^7^ CD33-CAR-NK cells or UTD-NK cells were administered via the tail vein and a daily subcutaneous injection of 25.000 IU IL-2 was started. As a control for tumor cell growth in vivo, one group of mice did not receive any treatment post tumor cell injection. The appearance, behavior, and weight of the animals were monitored every 2–3 days and the tumor load was assessed via bioluminescence imaging (BLI). At different time points, mice were sacrificed and femurs, tibiae, and spleens were isolated. To analyze BM cells, bone epiphyses were removed and BM cells were flushed out. GFP-positive tumor cells or CD56 and CD33-CAR expression were analyzed using a BD FACSCelesta flow cytometer. An anti-CD56-BV786 antibody (Clone NCAM16.2, BD Biosciences) and a CD33-CAR Detection Reagent (containing a recombinantly expressed fusion protein consisting of the human CD33 extracellular domains and a specifically mutated human IgG1 Fc region, Miltenyi Biotec) followed by an anti-biotin-PE antibody (clone REA746, Miltenyi Biotec) were used to determine CD56 and CD33-CAR expression, respectively.

### Cytometric Bead Array of cell culture supernatants and mouse blood

Cytokine release of CAR-NK and UTD-NK cells co-cultivated with AML cell lines or primary AML cells as well as cytokine levels of blood from mice three days pre tumor cell injection and one day post therapy initiation (four days post tumor cell application) were determined using a BD Cytometric Bead Array (CBA; BD Biosciences) or MACSPlex Cytokine Assays (Miltenyi Biotec). Supernatants of co-cultures from cytotoxicity assays were frozen at −80 °C. For the analysis of mouse serum, blood was obtained by cheek vein penetration and collected into serum tubes. BD CBA Flex Sets were used to measure cytokine concentrations in supernatants or serum, including GM-CSF, IFN-γ, macrophage inflammatory protein (MIP)-1a and tumor necrosis factor (TNF)-α (BD Biosciences). The tests were carried out following the manufacturer’s instructions. Data were obtained using a BD FACSVerse Bioanalyzer. For data analysis the FCAP Array software (v3.0.1; BD Biosciences) was used.

### Chimerism analysis

Mouse whole blood was collected in EDTA microvette tubes after cheek vein penetration and DNA was extracted using the QIAamp Mini spin column kit (Qiagen, Hilden, Germany). DNA content of samples was determined with a NP80 nanophotometer (Implen, Munich, Germany). The proportion of human DNA was assessed by a quantitative real-time PCR approach specific for the human Albumin gen [26]. The ratio of human NK and AML-target cells was calculated by chimerism analyses of informative human STR-markers using the Powerplex16 kit marker panel (Promega, Madison, WI, USA) [27].

### Bone embedding and sectioning

Bones were harvested and incubated in a series of 4% PFA, EDTA- and sucrose-solutions before they were embedded in gelatin. 7 µm-sections of bones were generated on the CryoStarTM NX70 cryostat (Thermo Fisher Scientific, Waltham, MA, USA). NK cells were stained with a Nkp46 antibody (R&D Systems, Minneapolis, MN, USA). For staining of CD33-CAR-NK cells, bone sections were incubated with CD33-biotin over night at 4 °C followed by the addition of secondary streptavidin AF647 antibody (BD Biosciences). For nuclear counterstaining DAPI (Sigma-Aldrich, St. Louis, MO, USA) was used.

### Statistical analysis

For statistical analysis, a normal distribution for NK cell functionality was assumed due to the deployment of healthy donors as NK cell source. Concerning the mouse in vivo-experiments also a normal distribution can be assumed. Accordingly, data were analyzed by two-tailed, unpaired Student’s *t*-test or Mann–Whitney-test and defined as significant when *p* < 0.05. Statistical analysis was performed using GraphPad PRISM version 6–8 (GraphPad Software, Inc., San Diego, CA, USA) for experiments with three or more donors/animals.

## Results

### CD33-CAR-NK cells demonstrate potent in vitro activity against AML

**Fig. 1:**
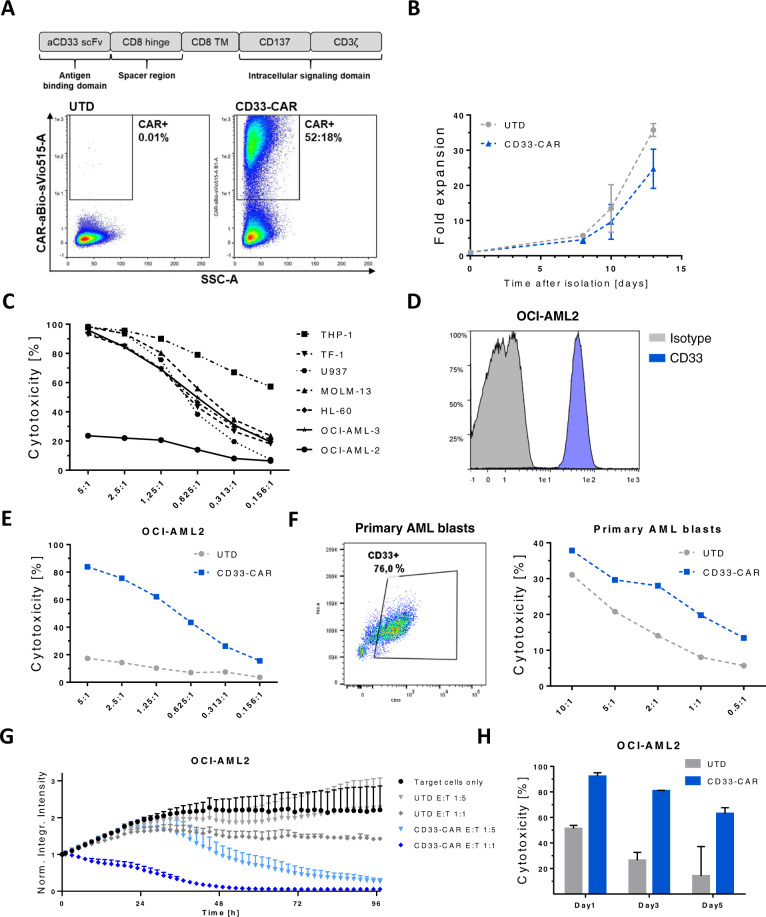
CD33-CAR-NK cells display robust in vitro effector function against CD33-positive AML cells that are partially resistant to natural cytotoxicity. **A** Schematic representation and surface expression of the CD33-directed second-generation CAR used in this study. Expression was analyzed by flow cytometry 12 days after transgene transfer into primary NK cells. **B** Time-lapsed expansion of CAR-transduced (CD33-CAR) and untransduced (UTD)-NK cells in the presence of IL-2 (500 IU/mL) and IL-15 (140 IU/mL) (*n* = 5). **C** Expanded NK cells show high cytotoxic activity against various AML cell lines except OCI-AML2. On day 14 of expansion, NK cells were co-incubated with various AML target cells at indicated E:T-ratios. After 24 h, the fraction of viable target cells was quantified by flow cytometry. Data shown are representative of results from two independent experiments. **D** The AML cell line OCI-AML2 displays high CD33 surface expression. **E**, **F** NK cells equipped with a CD33-CAR become highly cytotoxic against OCI-AML2 and CD33-positive primary AML cells. Cells were co-cultivated for 4 h and the viability of target cells was quantitated by flow cytometry. Two representative experiments are shown. **G** Dynamic monitoring of CAR-NK cell-mediated cytotoxicity. On day 12 after transduction, CAR-NK cells were co-cultured with (GFP^+^) OCI-AML2 cells and fluorescence emission was measured in the IncuCyte S3 imaging platform over 4 days. Shown is one representative from three separate experiments with a total of 5 donors. **H** Repetitive tumor-challenge assay revealed superior serial killing capacity of CD33-CAR-NK cells compared to UTD-NK cells. Expanded NK cells at day 12 post transduction were co-cultured with OCI-AML2 cells at an E:T-ratio of 1:1 and re-challenged with AML cells every other day. Shown is one representative experiment with a total of two donors. All graphs show mean of replicated ± SD.

Aiming to enhance the anti-leukemic activity of NK cells towards AML, primary NK cells derived from peripheral blood (PB) were genetically modified by lentiviral transduction to express a second-generation CD33-CAR using a single-chain variable fragment (scFv) derived from the clinically tested antibody-conjugate AVE9633 (Fig. 1A) [28]. Transgene integration by BaEV-LVs resulted in transduction rates between 35 and 60% and no severe impairment of expansion was observed during ex vivo cultivation, although CD33 has been reported to be upregulated on activated lymphocytes (Fig. 1A, B) [29]. For functional in vitro analysis, OCI-AML2 was selected as target-AML cell line, which showed relative insensitivity to natural cytotoxicity of NK cells and expressed CD33 (Fig. 1C, D). CD33-CAR-NK cells were more potent in eliminating OCI-AML2 and CD33-positive primary AML cells in a short-term killing assay compared to UTD-NK cells (Fig. 1E, F; Supplementary Fig. 1A), while killing of the CD33-dim KG1a cell line was not improved (Supplementary Fig. 1B). When the cytolytic activity was evaluated longitudinally by dynamic monitoring using IncuCyte, UTD-NK cells exhibited AML control at best, while CD33-CAR-NK cells efficiently eradicated OCI-AML2 cells even at the unfavorable E:T-ratio of 1:5 (Fig. 1G). Importantly, the sustained serial killing capacity of CD33-CAR-NK cells was also observed when the immune cells were re-challenged with AML cells supplied with fresh medium, indicating that the tumor cell death was not mediated by secondary mechanisms (for example increased levels of IFN-γ or TNF-α) but by sustained CAR-directed cytotoxic activity (Fig. 1H). Cytokine secretion and phenotypical analysis revealed no major differences between UTD and CD33-CAR-NK cells, again pointing towards a CAR-dependent killing mechanism (Supplementary Fig. 1C, D).

### A single dose of CD33-CAR-NK cells shows effective clearance of leukemic cells in the majority of OCI-AML2-engrafted NSG-SGM3 mice

**Fig. 2:**
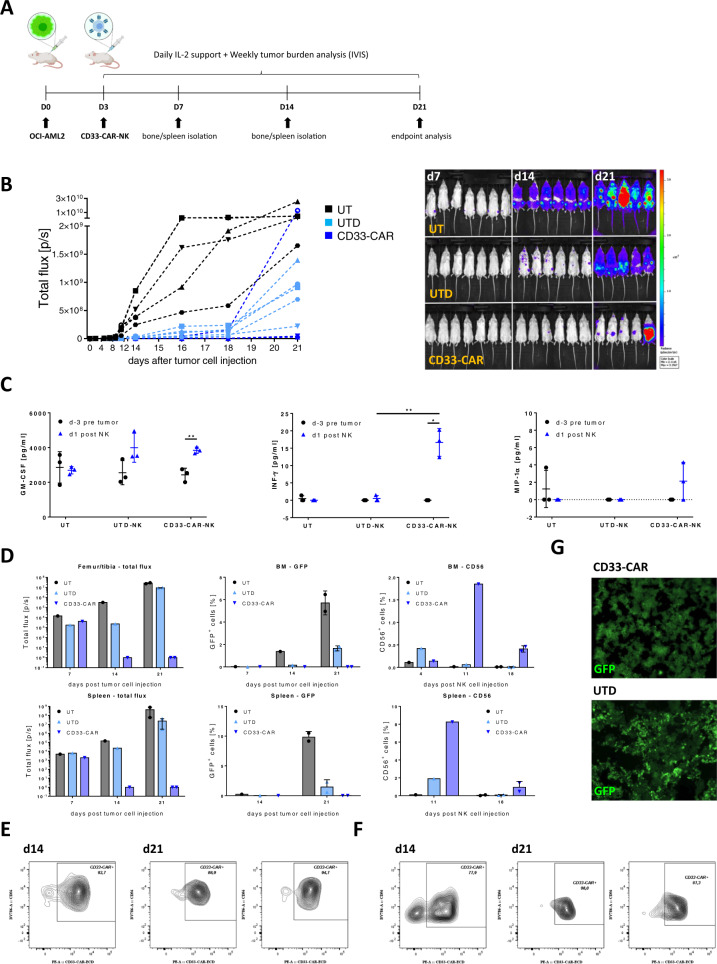
A single dose of CD33-CAR-NK cells displays potent anti-tumor efficacy in OCI-AML2 engrafted NSG-SGM3 mice. **A** Scheme of the in vivo evaluation of a single treatment with CD33-CAR-NK cells (1 × 10^7^ intravenously) followed by subcutaneous treatment with IL-2 in OCI-AML2 (Luc^+^) xenograft NSG-SGM3 mice. **B** Total flux analysis as well as representative BLI images of differently treated OCI-AML2 (Luc^+^) engrafted NSG-SGM3 mice over time (d7 *n* = 7; d14 *n* = 6; d21 *n* = 5 per group). Mice received a single dose of 1 × 10^7^ NK cells day 3 post AML cell injection. At day 21, 4 out of 5 mice (80%) that were treated with CD33-CAR-NK cells show severely reduced leukemic burden compared to untreated mice (UT) or mice which received untransduced (UTD)-NK cells. **C** Serum analysis of blood day 3 before AML injection and day 1 post first NK cell application shows significantly increased levels of GM-CSF as well as INF-γ for mice that had received CD33-CAR-NK cells (*n* = 3). Mean ± SD. **D** Total flux analysis of femurs/tibiae and spleens, as well as flow cytometry analysis of isolated cells from BMs or spleens at day 7, 14, and 21 post tumor cell injection, revealed the absence of GFP-positive tumor cells in CD33-CAR-NK-treated mice as well as increased NK cell infiltration (day 7/14 *n* = 1; day 21 *n* = 2 per group). Values of zero were set to 1 for total flux analysis. Median ± range. Flow cytometry-based CAR expression analysis of BM- (**E**) or spleen- (**F**) infiltrating NK cells at day 14 and 21 revealed the presence of mainly CAR-positive cells (day 14 *n* = 1; day 21 *n* = 2 per group). Mean ± SD. **G** Confocal microscopy imaging shows GFP-positive leukemia cells in BM of UTD-NK treated NSG-SGM3 mice at day 21 while absent in mice that received CD33-CAR-NK cells. Images from one representative animal are shown. Statistical analysis was performed by Student’s *t* test (*P < 0.05, **P < 0.01, ***P < 0.001, ****P < 0.0001).

With robust in vitro data in hand, the anti-leukemic effect of a single dose of CD33-CAR-NK cells was further analyzed in a humanized OCI-AML2 xenograft NOD.Cg-*Prkdc*^*scid*^ *Il2rg*^*tm1Wjl*^ Tg(CMV-IL3,CSF2,KITLG)1Eav/MloySzJ (NSG-SGM3) mouse model (Fig. 2A). This model was chosen, as NSG-SGM3 mice constitutively produce IL-3, GM-CSF, and stem cell factor (SCF), which reflects physiological conditions of a human host and promotes the engraftment of AML cells [25, 30]. NSG-SGM3 mice (male, 9–13 weeks old) received 0.5 × 10^6^ OCI-AML2 cells (GFP^+^, Luciferase (Luc)^+^) intravenously followed by a single administration of 1 × 10^7^ CD33-CAR-NK cells at day 3 post tumor cell injection. Remarkably a nearly complete eradication of leukemic cells by day 21 post injection of AML cells could be observed in the majority of mice (4 out of 5 mice at day 21 (80%)) while only a deceleration of leukemic cell growth could be observed in mice treated with UTD-NK cells (day 7: *n* = 7, day 14: *n* = 6, day 21: *n* = 5 per group; Fig. 2B). Quality control of applied CD33-CAR-NK cells revealed sufficient expansion (approximately 200-fold by day 20), high CAR- (60–80%), and CD16- (40–80%) expression as well as improved cytotoxic efficacy of the CD33-CAR-NK product (Supplementary Fig. 2A–D). Analysis of peripheral blood from CD33-CAR-NK treated mice revealed significant increase of human INF-γ serum levels on day 1 after the first NK cell-application compared to mice that had received UTD-NK cells, while serum levels of MIP-1α, IL-6, IL-10, and TNF-α remained below physiological relevant concentrations (Fig. 2C; Supplementary Fig. 2E). BLI analysis of femur, tibia, and spleen of representative mice on day 7, 14, and 21 revealed impeded AML engraftment in CD33-CAR-NK treated animals which was confirmed by flow cytometry analysis of cells isolated from BM and spleen (Fig. 2D). Furthermore, CD33-CAR-NK cells showed increased BM- and spleen-infiltration compared to UTD-NK cells. Thereof, the majority of NK cells could be identified as CAR-positive (BM: 89–95%; spleen: 78–90%; Fig. 2E, F). Consequently, BM-located CD33-CAR-NK cells prevented engraftment of leukemic cells and the successive alteration of the BM, which became evident not only by flow cytometry but also through histologic analysis of BM via confocal microscopy. Thereby, BM of untreated mice or mice which had received UTD-NK cells displayed an altered, pitted structure together with high GFP-positive AML cell infiltration, while a normal BM structure and no GFP signal could be observed for mice treated with CD33-CAR-NK cells (Fig. 2G).

### Enhanced clearance of AML in OCI-AML2-engrafted NSG-SGM3 mice by repetitive injections of CD33-CAR-NK cells

**Fig. 3:**
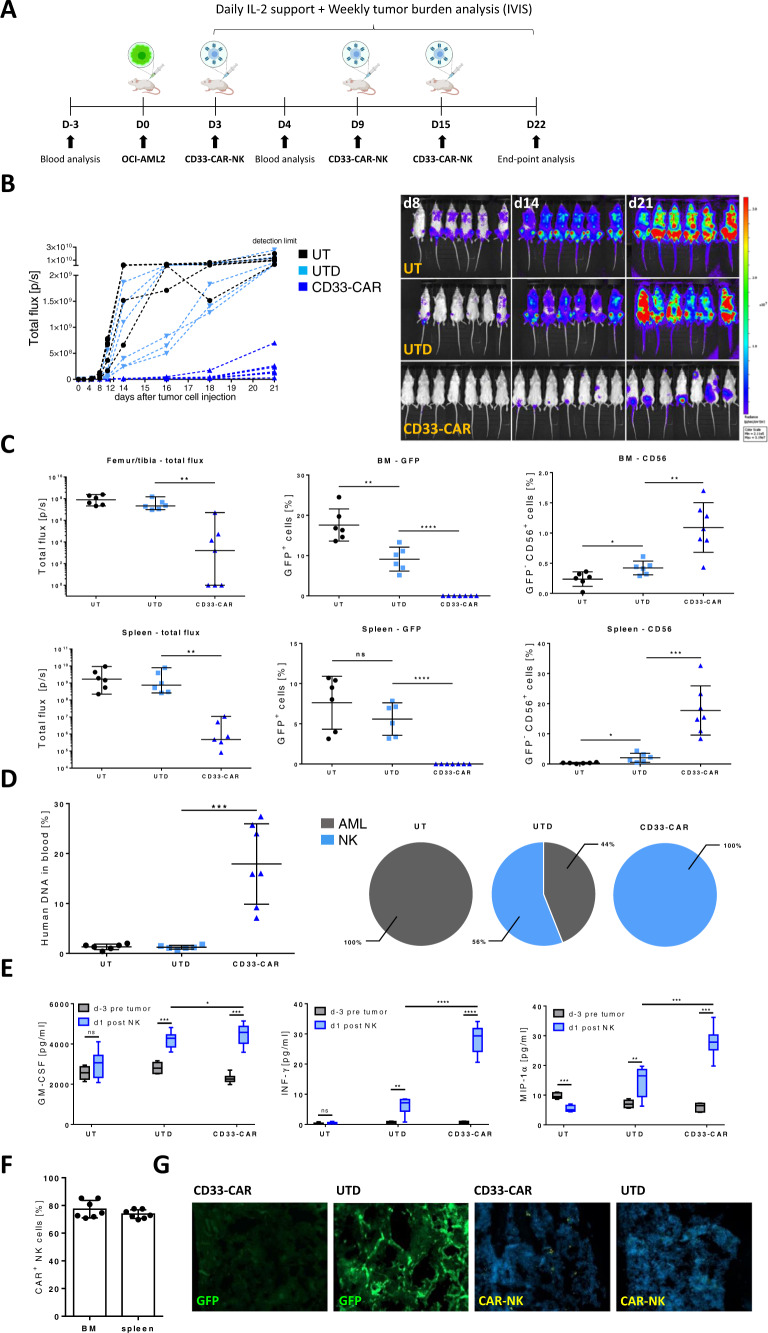
Repetitive administration of CD33-CAR-NK cells displays improved anti-tumor efficacy in OCI-AML2 engrafted NSG-SGM3 mice. **A** Scheme of the in vivo evaluation of a repetitive treatment with CD33-CAR-NK cells (1 × 10^7^ intravenously) combined with subcutaneous IL-2 treatment in OCI-AML2 (Luc^+^) xenograft NSG-SGM3 mice. **B** Total flux analysis, as well as representative BLI images of differently treated OCI-AML2 (Luc+), engrafted NSG-SGM3 mice over time (*n* = 7 per group). Mice received a total of three weekly doses of 1 × 10^7^ NK cells. Mice that were treated with CD33-CAR-NK cells show severely reduced leukemic burden compared to untreated mice (UT) or mice which received untransduced (UTD)-NK cells. **C** Total flux analysis of femurs/tibiae and spleens, as well as flow cytometry analysis of isolated cells from BMs or spleens at day 22 post AML-injection, revealed the absence of GFP-positive leukemic cells in CD33-CAR-NK treated mice as well as increased NK cell infiltration (*n* = 6–7 per group). Values of zero were set to 1 for total flux analysis. Median ± range. **D** Chimerism analysis d22 post AML-injection revealed high amounts of DNA from human NK cells without detectable DNA of AML in blood of mice that were treated with CD33-CAR-NK cells (*n* = 6–7 per group). Mean ± SD. **E** Serum analysis of blood day 3 before AML injection and day 1 post first NK cell application showed significantly increased pro-inflammatory human cytokines for mice that received CD33-CAR-NK cells (*n* = 6–7 per group). **F** Flow cytometry-based CAR-expression analysis of BM- or spleen-infiltrating NK cells in CD33-CAR-NK treated mice revealed the presence of mainly CAR-positive cells (*n* = 6–7 per group). Mean ± SD. **G** Confocal microscopy imaging demonstrated GFP-positive leukemia cells in BM of UTD-NK treated NSG-SGM3 mice while absent in mice that received CD33-CAR-NK cells. Additionally, CAR-NK cells could be detected in the BM of CD33-CAR-NK treated mice. One representative image from a total of four are shown. Statistical analysis was performed by Mann–Whitney-test (for total flux analysis) or Student’s *t* test (for the rest) (*P < 0.05, **P < 0.01, ***P < 0.001, ****P < 0.0001).

With the aim of improving outcomes, following CAR-NK-product characterization (Supplementary Fig. 3A–C), in total three doses of 1 × 10^7^ CD33-CAR-NK cells were administered to a group of seven OCI-AML2-engrafted NSG-SGM3 mice (female, 9–13 weeks old) (Fig. 3A). This treatment resulted in strong reduction of AML burden by day 21 in all animals (Fig. 3B). BLI analysis of femurs, tibiae, and spleens on day 22 revealed impeded AML engraftment in CD33-CAR-NK-treated mice while the administration of UTD-NK cells did not have any effect. This was confirmed by flow cytometry analysis of cells isolated from BM and spleen. Here only a reduction of BM- and no changes for spleen-infiltrating AML cells could by observed in mice treated with UTD-NK cells, whereas CD33-CAR-NK cells were able to completely eradicate AML cells in both organs (Fig. 3C). Additionally, NK cell-infiltration in the BM or spleen on day 22 was significantly increased in mice treated with CD33-CAR-NK compared to UTD-NK cells, and the majority of NK cells were identified as CAR-positive (78–95%; Fig. 3F). Chimerism analysis of PB revealed high amounts of DNA from human NK cells (10–30%) without detectable DNA of human AML cells in mice, which were treated with CD33-CAR-NK cells. In contrast, only small amounts of human DNA were found in the blood samples of mice treated with UTD-NK cells which partially comprised AML cell-DNA (Fig. 3D). Auxiliary, significant increases in human INF-γ and MIP-1α serum levels on day 1 after the first NK cell-application could be observed in mice, which had received CD33-CAR-NK compared to UTD-NK cells, while serum levels of IL-6, IL-10, and TNF-α remained below physiological relevant concentrations (Fig. 3E; Supplementary Fig. 3E). Histologic analysis of BM by confocal microscopy of mice treated with CD33-CAR-NK cells revealed the presence of CAR-NK cells in an intact, AML-free BM, while UTD-NK-treated or untreated mice displayed an altered, pitted BM structure together with high GFP-positive AML cell infiltration (Fig. 3G; Supplementary Fig. 3D).

Of note, following both single and repetitive CD33-CAR-NK cell injections, the animals did not show any clinical signs of cytokine release syndrome (CRS), weight reduction, altered appearance or behavior, or graft-versus-host-disease (GvHD), in line with histological analysis of lung, liver, and colon.

## Discussion

In the rapidly moving field of CAR-immune cell therapies, recent studies have shown that primary CAR-NK cells are safe and can be highly effective [31]. With the aim of developing NK cell therapy concepts that address malignant diseases beyond CD19-expressing B cell neoplasia, which are still difficult to treat, research on primary CAR-NK cells targeting AML is urgently required. Our study showed that CD33-CAR-NK cells exhibit stable CAR expression and maintained a high proliferation rate, although fratricide of CD33-positive NK cells was observed in different donors (Supplementary Fig. 1E, F). Importantly, CD33-CAR-NK cells efficiently eliminated AML cells in vitro and eradicated all BM- and spleen-infiltrating as well as the majority of peripheral AML cells in NSG-SGM3 mice without observable side effects. Significantly higher numbers of BM-, spleen- as well as PB-NK cells were detected in mice treated with CD33-CAR-NK cells. Additionally, while the CAR-expression rates of the applied NK cells reached between 30 and 50%, the majority (≥80%) of BM- and spleen-infiltrating NK cells could be identified as CAR-positive. This indicates increased survival or improved BM homing of CAR-NK cells. Latter constitutes a particularly desirable feature as the density of BM located NK cells following adoptive NK cell-transfer was shown to correlate with clinical response [32]. The increased persistence of CD33-CAR-NK cells compared to UTD-NK cells without endogenous cytokine-production might also constitute a clinical advantage, as recent studies showed that CAR-NK cells constitutively secreting IL-15 can cause severe toxicities in animal models [33]. Additionally, increased but still moderate values of pro-inflammatory cytokines in mice treated with CD33-CAR-NK cells could fortify a systemic immune response against AML, which might further improve outcomes. While donor-dependent heterogeneity in terms of CAR-expression and proliferation constitutes a known hurdle in vitro, we could also observe strong donor-dependent differences in anti-leukemic effects of UTD-NK cells in vivo, which was mostly abrogated by introducing a CAR-dependent killing mechanism. Nevertheless, donor-dependent characteristics of allogeneic applied NK cells need to be carefully monitored and require further investigations to maximize outcomes and avoid unwanted side effects. While in recent studies with CD33-CAR-T cells, certain constructs such as Gemtuzumab-based CARs were shown to induce in vivo toxicities, the AVE9633-derived CAR deployed in this study might pose a promising option for AML therapy [34]. Overall, these data clearly suggest that CD33-CAR-NK cells might be suitable for the treatment of AML, due to their strong anti-tumor efficacy and highly improved presence in BM, spleen, and PB compared to normal NK cells. In particular, for cases in which the generation of autologous CAR-immune cells from heavily pre-treated AML patients appears to be difficult, CAR-NK products from third-party donors may be considered a clinically important advantage.

## Supplementary information

Supplemental Figures and legends
