# Supplementary material for: Primary CD33-targeting CAR-NK cells for the treatment of acute myeloid leukemia
Source: Blood Cancer J. 2022 Apr 13;12(4):61. doi: 10.1038/s41408-022-00660-2 (PMC9007937; doi:10.1038/s41408-022-00660-2)
Supplement: Supplementary file 1 — Supplemental Figures and legends [file 41408_2022_660_MOESM1_ESM.pdf]

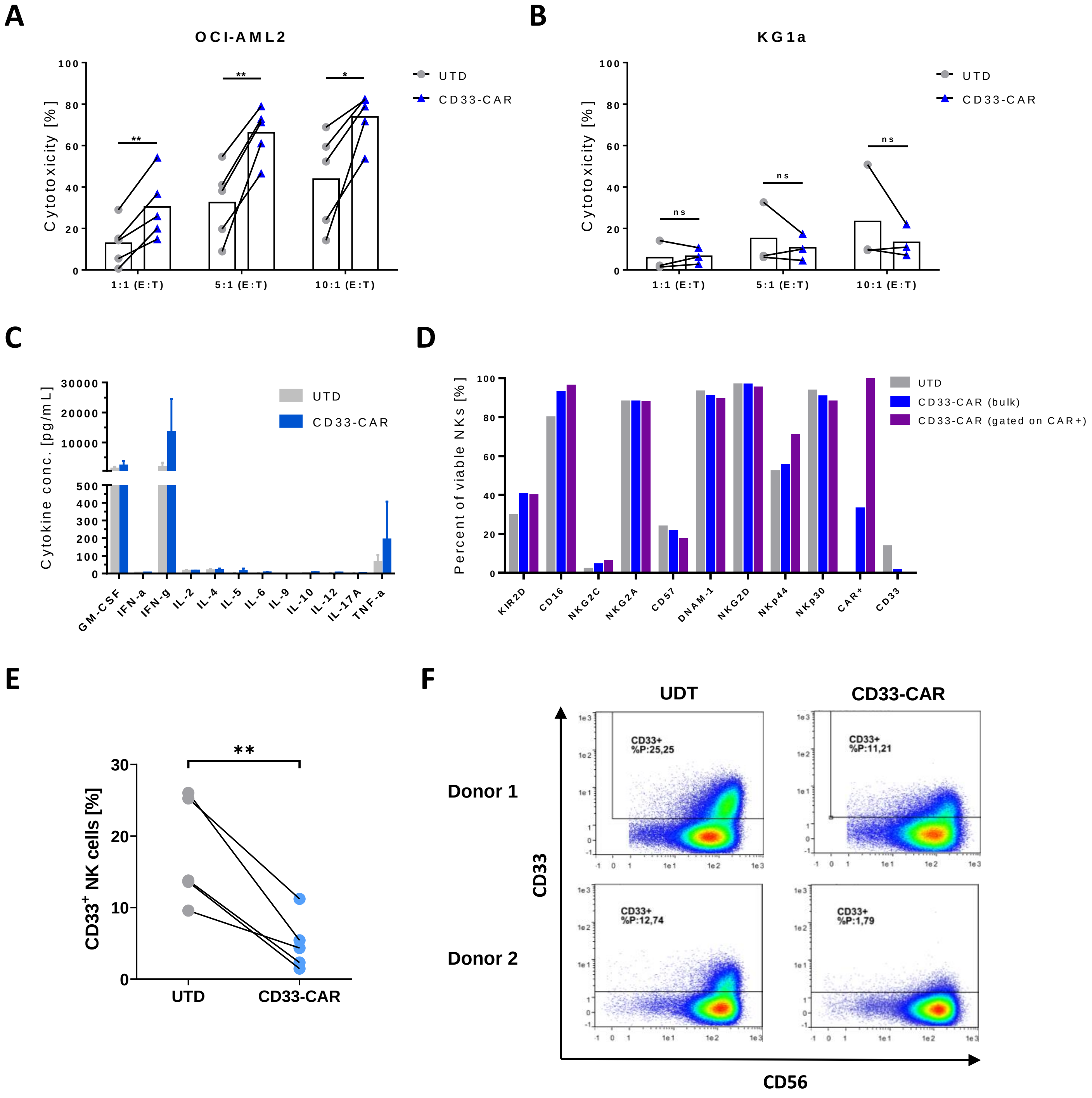

Supplementary Figure 1

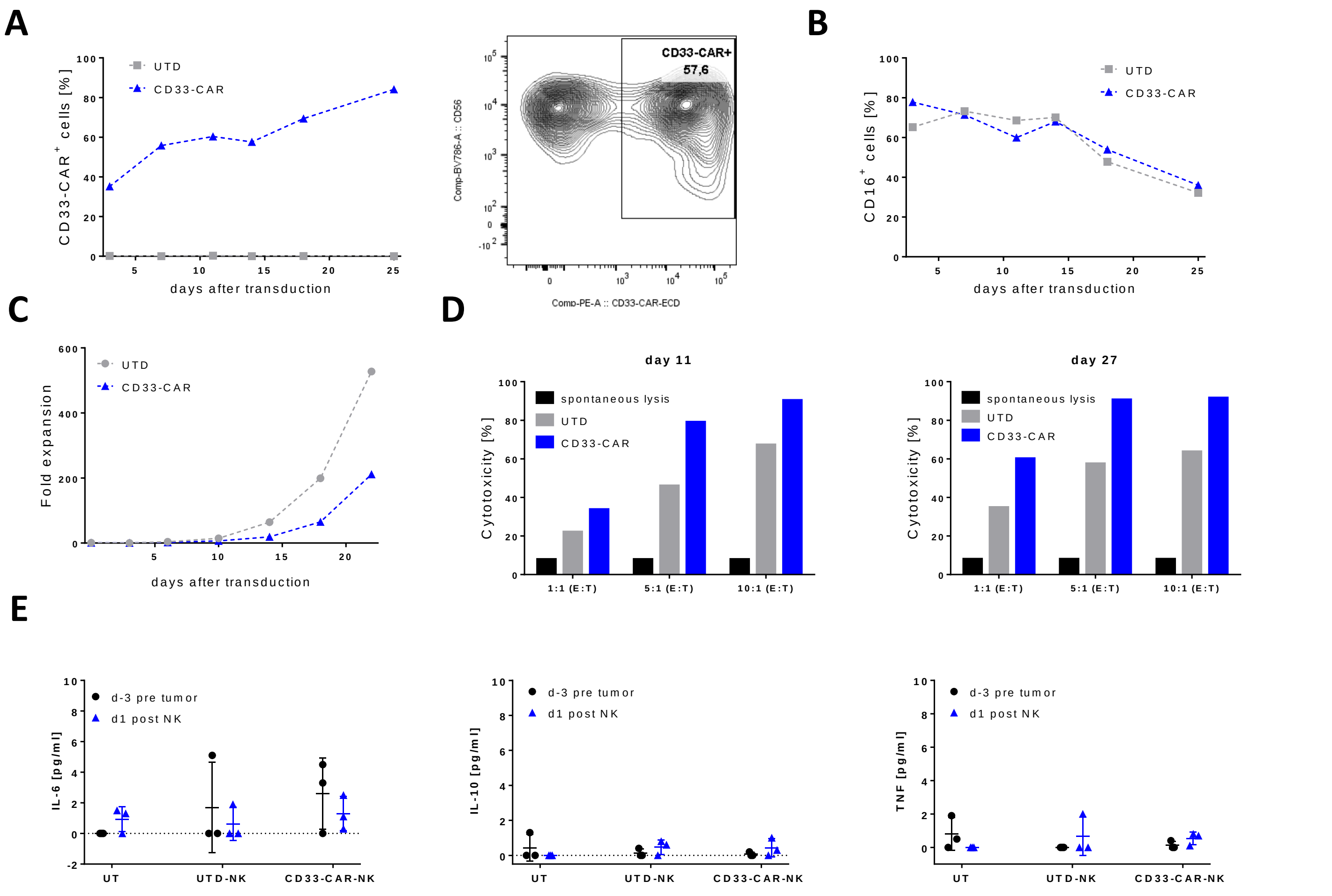

Supplementary Figure 2

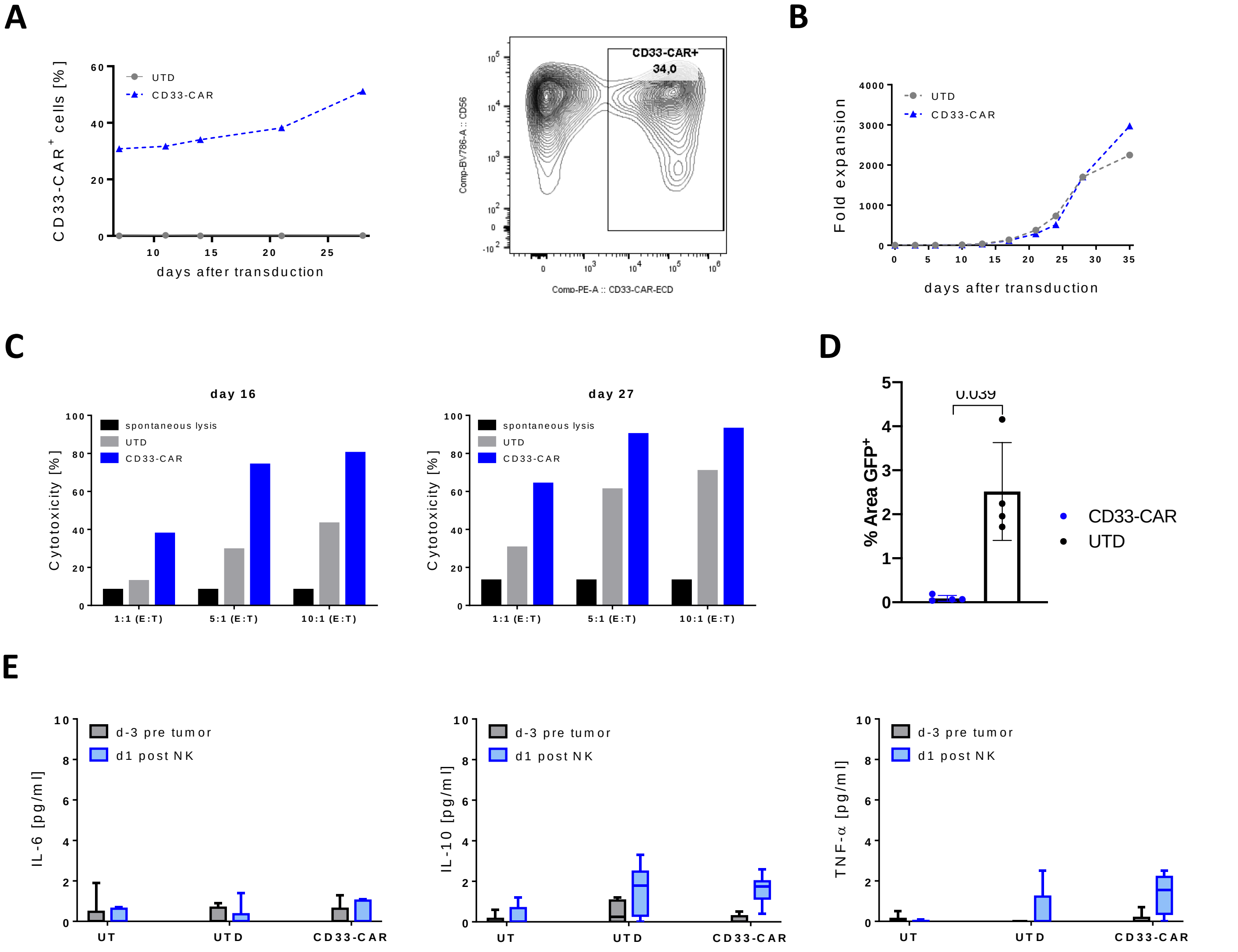

Supplementary Figure 3

Legends to supplementary figures

Supplementary Figure 1: CD33-CAR-NK cells display robust in vitro effector function against CD33-positive AML cells that are partially resistant to natural cytotoxicity. A CD33-CAR-NK cells display increased cytotoxicity against CD33-positive OCI-AML2 cells (**B**) while no difference could be observed against the CD33-dim KG1a cell line. Cells were co-cultivated for 4 hours and the viability of target cells was quantitated by flow cytometry. Connected dots represent individual experiments (n=3-5). Bars represent means. **C** Cytokine secretion profile of CD33-CAR-NK cells vs. untransduced (UTD)-NK cells following co-culture with OCI-AML-2 cells (E:T-ratio, 1:1). On day 12 post transduction, CD33-CAR-NK cells were co-cultured with target cells for 24 hours and culture supernatants were analyzed for cytokine secretion using the MACSPlex technique (n=4). Means ± SD. **D** Flow cytometry-based phenotypical analysis showed no difference in maturation and activation state of transduced NK cells. Data shown are from one representative experiment with a total of two donors. CD33 expression in CAR-NK cells was not specified due to the overall low CD33 expression on CD33-CAR-NK cell bulk. **E** The CD33 expression of UTD or CD33-CAR-NK cells was determined at day 13 of culture by flow cytometry analysis (n=5). **F** Exemplary dot plots of the CD33 expression on NK cells from two donors are shown. Statistical analysis was performed by student's *t* test (*P <0.05, **P<0.01).

Supplementary Figure 2: A single dose of CD33-CAR-NK cells displays potent anti-tumor efficacy in OCI-AML2 engrafted NSG-SGM3 mice. A-D In vitro analysis of applied CAR-NK cells show stable, high CAR expression, similar CD16 expression compared to UTD-NK cells, slightly attenuated proliferation and improved cytotoxicity against OCI-AML2 cells at day 11 and day 27 post transduction. For cytotoxicity assays technical triplicates were measured. Mean ± SD. **E** Analysis of blood day 3 pre tumor cell injection and day 1 post first

NK cell application shows only minor changes in serum levels of IL-6, IL-10 and TNF-α (n=3). Statistical analysis was performed by student's *t* test.

Supplementary Figure 3: Repetitive administration of CD33-CAR-NK cells display improved anti-tumor efficacy in OCI-AML2 engrafted NSG-SGM3 mice. A-C Applied CAR-NK cells show stable, high CAR expression, slightly attenuated proliferation and improved cytotoxicity against OCI-AML2 cells at day 16 and day 27 post transduction. For cytotoxicity assays technical triplicates were measured. Mean ± SD. **D** Histologic analysis of BM confirms the absence of tumor cells in CD33-CAR-NK treated mice while high tumor loads are present in mice which received UTD-NK cells (n=4). Mean ± SD. **E** Analysis of blood day 3 pre tumor cell injection and day 1 post first NK cell application shows only minor changes in serum levels of IL-6, IL-10 and TNF-α (n=6-7). Statistical analysis was performed by student's *t* test.
